# *chi* sequences switch the RecBCD helicase–nuclease complex from degradative to replicative modes during the completion of DNA replication

**DOI:** 10.1016/j.jbc.2023.103013

**Published:** 2023-02-11

**Authors:** Nicklas A. Hamilton, Avery E. Jehru, William N. Samples, Brian M. Wendel, Parisa D. Mokhtari, Charmain T. Courcelle, Justin Courcelle

**Affiliations:** 1 Department of Biology, Portland State University, Portland, Oregon, USA; 2 Department of Microbiology, Cornell University, Ithaca, New York, USA

**Keywords:** DNA replication, replication completion, RecBCD, *chi*, genome stability, 2D, 2-dimensional, *chi*, crossover hotspot instigator sequence, *ter*, termination sequence

## Abstract

Accurately completing DNA replication when two forks converge is essential to genomic stability. The RecBCD helicase–nuclease complex plays a central role in completion by promoting resection and joining of the excess DNA created when replisomes converge. *chi* sequences alter RecBCD activity and localize with crossover hotspots during sexual events in bacteria, yet their functional role during chromosome replication remains unknown. Here, we use two-dimensional agarose gel analysis to show that *chi* induces replication on substrates containing convergent forks. The induced replication is processive but uncoupled with respect to leading and lagging strand synthesis and can be suppressed by *ter* sites which limit replisome progression. Our observations demonstrate that convergent replisomes create a substrate that is processed by RecBCD and that *chi*, when encountered, switches RecBCD from a degradative to replicative function. We propose that *chi* serves to functionally differentiate DNA ends created during completion, which require degradation, from those created by chromosomal double-strand breaks, which require resynthesis.

The mechanism by which cells complete DNA replication at the precise point where two replication forks converge is essential to maintain genome stability and can result in amplifications, deletions, and a loss of viability when the process is impaired. To complete replication accurately, cells encode an enzymatic system that is capable of recognizing or counting in pairs and joins the nascent strands of converging replication forks at the point where all sequences have precisely doubled. A failure to complete any single event where replication forks converge would be expected to result in a loss of genomic stability, mutation, or cell lethality. Yet, this reaction occurs thousands of times per generation along the chromosomes of human cells and must occur with remarkable efficiency. Given this critical role and considering the large number of proteins that cells devote to ensure fidelity during replication initiation and elongation, it is not surprising that this final step has been found to involve several enzymes devoted to this tightly regulated reaction (1, 2, 3, 4, 5, 6, 7, 8, 9, 10, 11, 12, 13).

Unlike human cells, the completion of replication in *Escherichia coli* occurs only once each cell cycle, typically within a ∼400-kb region of the chromosome that is opposite to its bidirectional origin of replication (14, 15, 16, 17, 18). This region is flanked by *ter* sequences which block replisome progression in an orientation-specific manner (17). *ter* is a nonpalindromic, 23 bp consensus sequence which binds to the protein Tus. When replication approaches in the nonpermissive direction, strand unwinding flips the sixth base of the consensus sequence (C6) into a pocket on Tus, locking the protein onto the DNA (19). Although *ter* ensures that completion occurs within this region, it is not involved in the reaction, as chromosomes lacking *ter* replicate normally and are stably maintained (20, 21, 22).

Current models suggest that the processivity of replicative helicases leads to replisomes partially bypassing each other at the point where they converge. This creates a limited region of over-replicated DNA containing three copies of the genetic information. The completion reaction initiates through the action of structure-specific nucleases SbcCD and ExoI, which incise and resect the palindrome-like intermediate created by convergent replisomes (7, 9, 10, 11). In the absence of the SbcCD and ExoI nucleases, these over-replicated regions persist. Growth and viability in these cells become dependent on RecA, which then resolves the chromosomes through an aberrant form of recombination. Under these conditions, the excess regions of DNA are not degraded, resulting in genetic instabilities and amplifications at these loci (9, 10, 11). Similar instabilities and amplifications are observed in eukaryotic cells lacking the homologs, Mre11-Rad50 and Sae2, arguing that completion is highly conserved throughout evolutionarily diverged organisms (23, 24, 25).

RecB, RecC, and RecD form a dual helicase–nuclease complex that plays a critical role in completing replication (8, 9, 26, 27, 28, 29). Following incision by SbcCD, RecBCD activity is required to process the over-replicated region and catalyze or recruit enzymes that promote joining of the convergent strands (8, 9, 10, 11). *In vitro*, this complex is highly processive, capable of unwinding and degrading 1000 to 2000 base pairs per second, a rate that approximates the ability of replisomes to synthesize DNA during replication (27, 30, 31). Loss of RecB or RecC inactivates the enzyme complex. However, loss of RecD retains the helicase activity while inactivating the nucleolytic degradation (27, 31, 32). In *recB* or *recC* mutants, the failure to join the nascent ends of convergent replication forks leads to excessive degradation at these loci on the chromosome, severely compromising growth and viability in these cultures (2, 8, 9). In the absence of RecD, degradation of the excess sequence is partially impaired. However, nascent end joining appears to occur normally, and viability is not compromised (8, 9, 10).

*chi* (crossover hotspot instigator) sequences, 5′GCTGGTGG3′, alter the activity of the RecBCD complex. During recombinational processes, these sequences are associated with loci where crossovers are joined to form recombinant molecules during sexual events (31, 33, 34, 35, 36, 37, 38). During unwinding, the 8 bp *chi* sequence is recognized by the RecC subunit of the complex, inducing a conformational change that brings the nuclease domain of RecB proximal to the single strand DNA as it exits the RecC subunit, allowing incision and creating a 3′ single strand end upon continued unwinding (39). Following encounters with a *chi* sequence, further nucleolytic activity is then attenuated while RecB helicase activity is maintained, similar to what is seen in complexes lacking RecD (38, 40, 41). *chi* sequences are highly over-represented in the chromosome and, intriguingly, their presence is heavily biased on the leading strand of replication (9, 42, 43). The purpose of this strand bias is difficult to reconcile with classical models of double-strand break repair, since a break would have two double-strand ends with both *chi*-enriched and -nonenriched strands and suggests a role associated with chromosome replication.

Plasmid minichromosomes, which contain a bidirectional origin of replication, have been used to effectively characterize aspects of how converging forks complete replication *in vivo* (11). Unlike plasmids with unidirectional origins, plasmid minichromosomes require RecBCD to propagate in cells, supporting the idea that these enzymes act specifically to process a structure created when two replisomes converge (11). Additionally, similar to the chromosome, genetic instabilities and amplifications on minichromosome plasmids are driven by an aberrant, RecA-mediated recombinational reaction (11). Given RecBCD’s role in completing DNA replication, we considered whether *chi* sequences may also play a role in this reaction. We show that *chi* induces replication on substrates that contain convergent replisomes, leading to amplifications and multimeric structures that destabilize the minichromosome. The replication can be limited, and the destabilization can be suppressed by the addition of *ter* sequences placed in an orientation similar to that occurring in the terminus of the chromosome.

## Results

### Convergent replication forks require RecBCD processing

**Figure 1:**
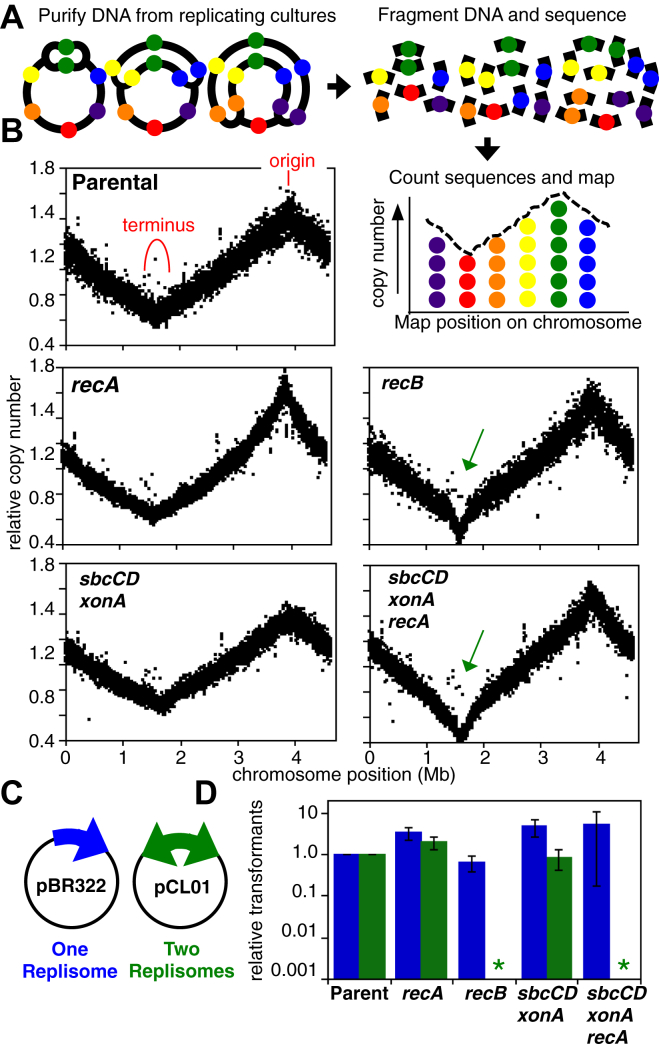
**The ability to maintain the chromosome region where replication completes correlates with the ability to maintain two-replisome plasmids**. *A*, a diagram of the method employed to profile replication on the chromosome. Genomic DNA from replicating cultures is purified, fragmented, and profiled using high-throughput sequencing. *B*, in both wildtype cultures and *recA* mutants, replication proceeds bidirectionally from the origin and completes in the terminus region, as indicated. *recB* mutants and *sbcCD xonA* mutants in the absence of RecA are unable to maintain the chromosome region where replication completes (*green arrow*). Sequence read frequencies at each chromosome loci, normalized to stationary phase cells, are plotted. *C*, diagram of the one-replisome and two-replisome plasmids used to transform each strain. *D,* the transformation frequency, relative to wildtype cells, is shown for each strain indicated. Error bars represent the standard error of three independent experiments. ∗Indicates transformants were below the assay's limit of detection

The ability to complete replication can be examined by profiling replication across the chromosome. In this technique, genomic DNA from replicating cultures is first purified, fragmented, and sequenced using high-throughput sequencing. Then, the number of sequences that align to each segment of the chromosome is counted and plotted (Fig. 1*A*). In rapidly growing cultures, sequences proximal to the bidirectional origin, which replicates first, are observed at the highest frequency. Sequence frequencies then decrease inversely with their distance from the origin, until reaching the region where the two forks converge and replication completes as shown in our parental strain (Fig. 1*B*). The profile of *recA* mutants looks similar to the parental strain, demonstrating that completion occurs independently of RecA or homologous recombination. In contrast, a loss of sequences is observed in the region where replication forks converge in *recB* mutants, and similar results have been reported for mutants lacking both *recB* and *recC*, as well as *recB*, *recC*, and *recD* (8, 10). The frequency of terminus region sequences is reduced approximately twofold in *recB* cells relative to wildtype cultures.

Considering that greater than half of all sequence reads from a culture must correspond to the parental DNA strands, one can infer that nearly all *recB* mutant cells are unable to maintain this region of the chromosome (8, 10). The observations indicate that the replication defect in *recBCD* mutants is specific to the region where replication forks converge. The independence of this process from RecA activity argues that unlike double-strand repair, the function of RecBCD in completing replication likely involves an intramolecular rather than intermolecular reaction. The inability to maintain the terminus region of the chromosome severely impairs growth and viability in these mutants (8, 10). Completion initiates through the action of the structure-specific nucleases, SbcCD and ExoI. In cultures lacking these enzymes, growth and viability is maintained by RecA and an aberrant form of recombination (10). In the absence of RecA, *sbcCD xonA* cells are unable to maintain the terminus region of the chromosome, which compromises growth and viability, similar to that seen *recBCD* cultures (Fig. 1*B*).

We next examined whether a cell’s ability to maintain plasmids containing two replisomes correlates with its ability to maintain the terminus region of the chromosome. A mixture of pCL01, which utilizes a bidirectional origin, and pBR322, which uses a unidirectional origin were transformed into each strain, and the number of transformants was counted following overnight incubation on plates specifically selecting for each plasmid (Fig. 1*C*). Whereas, the one-replisome plasmid could be transformed and maintained in all strains, transformation frequency of the two-replisome plasmid was severely reduced in both *recB* and *sbcCD xonA recA* mutants (Fig. 1*D*). These enzymatic requirements mimic the conditions needed to maintain the region of the chromosome where replication forks converge.

Taken together, the results demonstrate that two-replisome plasmids can be used to characterize the completion reaction and that RecBCD is required to process a substrate specifically created when two replication forks converge.

### *chi* sequences induce replication on substrates containing convergent replication forks

**Figure 2:**
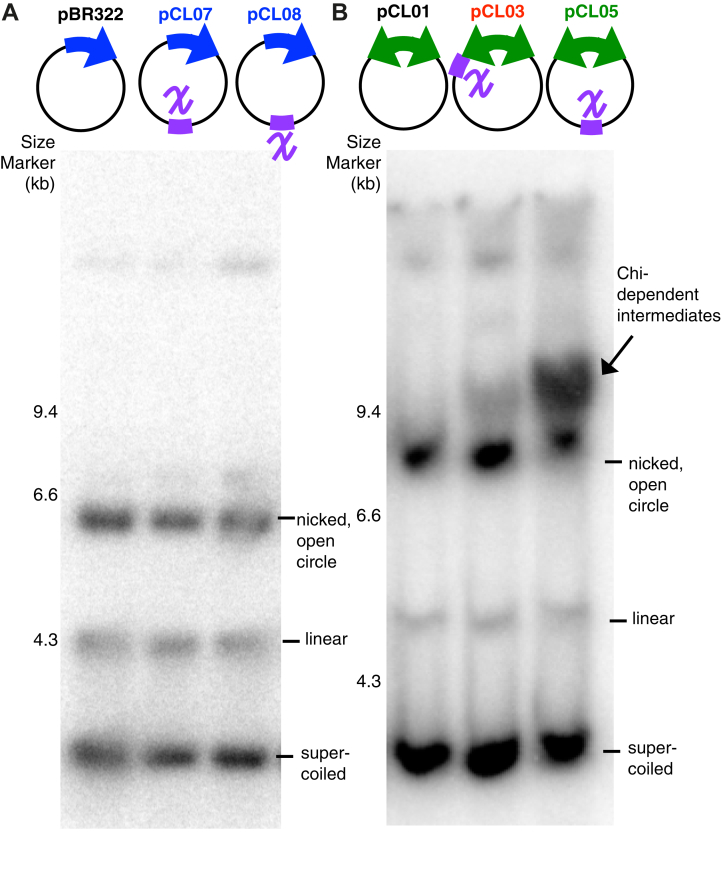
**The presence of *chi* induces replication intermediates on plasmids that contain two replisomes, but not one replisome**. *A*, on plasmids containing one replisome, no effect of *chi* is observed. Lane 1, no *chi*; lane 2, *chi* in the leading-strand template; lane 3, *chi* in the lagging-strand template. *B*, on plasmids containing two replisomes, additional replication intermediates are observed when *chi* sequences are present. Lane 1, no *chi*; lane 2, *chi* located proximal to the origin; lane 3, *chi* located opposite to the origin. Total DNA, genomic and plasmid, was purified and electrophoresed in 1% agarose gel and analyzed by Southern analysis using ^32^P-labeled pBR322 (for *A*) or pCL01 (for *B*) as a probe. The migration position of supercoiled, linear, and nicked, *open circle* plasmid is indicated. The migration position of the *chi*-induced intermediate is indicated with an *arrow*. Size marker, lambda Hind III-digested DNA.

To examine how *chi* affects the completion reaction, we engineered this sequence into the two-replisome plasmid at loci proximal (pCL03) and distal (pCL05) to the origin of replication. As controls, we also engineered the *chi* sequence into the leading (pCL07) and lagging (pCL08) strand of the one-replisome plasmid. These plasmids were transformed in the parental strain and grown to mid-log phase before the total cellular DNA was purified. The genomic DNA was then electrophoresed through a 1% agarose gel, and the plasmid DNA was examined by Southern analysis using the corresponding [^32^P]-labeled plasmids as a probe (Fig. 2). When the one-replisome plasmid was used, no differences were seen on the plasmid molecules irrespective of the presence of *chi* sequences. However, when *chi* sequences were placed into the two-replisome plasmids, prominent, higher-order replication intermediates were observed. In the absence of *chi*, these intermediates were not observed. The presence of *chi*-induced replication specifically on plasmids containing two-replisomes suggests it may be associated with converging replication forks, such as occurs during the completion reaction on the chromosome.

**Figure 3:**
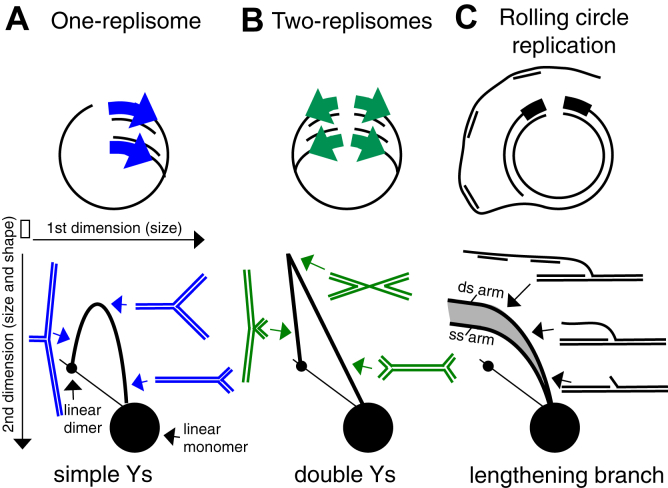
**Diagrams of the expected replication patterns observed in 2D agarose gels for DNA fragments containing one replisome, or two replisomes when digested with an enzyme that cuts the plasmid at its origin of replication**. The first dimension resolves molecules based primarily on size, where smaller molecules migrate more rapidly. The second dimension resolves molecules based primarily on the ‘awkwardness’ of shape, where nonlinear molecules migrate more slowly. Following restriction digestion, nonreplicating plasmids run as a linear monomer fragment, forming the prominent spot observed in gels. *A*, for plasmids with unidirectional origins, replicating fragments form Y-shaped molecules, forming an arch that extends out from the linear monomer fragment and returns to the linear dimer fragment. *B*, for plasmids with bidirectional origins, replicating fragments form double Y-shaped and X-shaped molecules. Double Y-shaped molecules form a line that extends out from the linear monomer fragment, while X-shaped molecules appear as a line extending up from the linear dimer fragment to a point where the two lines meet. *C*, if rolling circle replication occurs on either plasmid, the replicating fragments would form linear molecules having a single branch of varying lengths, migrating as a broad arc that extends out from the linear monomer fragment. Single-strand regions on the rolling circle product, caused by a lack of lagging strand synthesis would form a similar arc (ss-arm) but migrate slightly faster than a branch that contains entirely double-stand DNA (ds-arm).

To further characterize these intermediates, we examined the structural properties of replicating plasmids using 2-dimensional (2D) agarose gel analysis (44, 45, 46, 47). To this end, total genomic DNA was purified from growing *E. coli* cultures that contained each plasmid. The DNA was then digested with a restriction enzyme that cleaved the plasmids at their origin of replication, and the plasmid DNA was analyzed by 2D agarose gel electrophoresis. Nonreplicating plasmids migrate as a linear fragment forming the prominent spot observed on the 2D gel. Replicating fragments form structures that migrate more slowly because of their larger size and nonlinear shape. On plasmids containing a unidirectional origin, replicating fragments would be expected to form a simple Y-arc that extends out from the linear fragment following 2D gel electrophoresis (Fig. 3*A*). Plasmids with a bidirectional origin of replication would be expected to produce fragments with double Y-shapes, migrating in an inverted V-shape that extends up from the linear monomer fragment and down to the linear dimer fragment (Fig. 3*B*). DNA arising from rolling circle replication would produce a double-strand linear fragment with a branched DNA tail, migrating as a broad curve that rises out from the linear fragment (Fig. 3*C*). Any single-strand regions produced by the rolling circle replication would be resistant to digestion by the restriction endonuclease and migrate slightly faster than the double-strand arc, with the potential to form long, unrestricted fragments.

**Figure 4:**
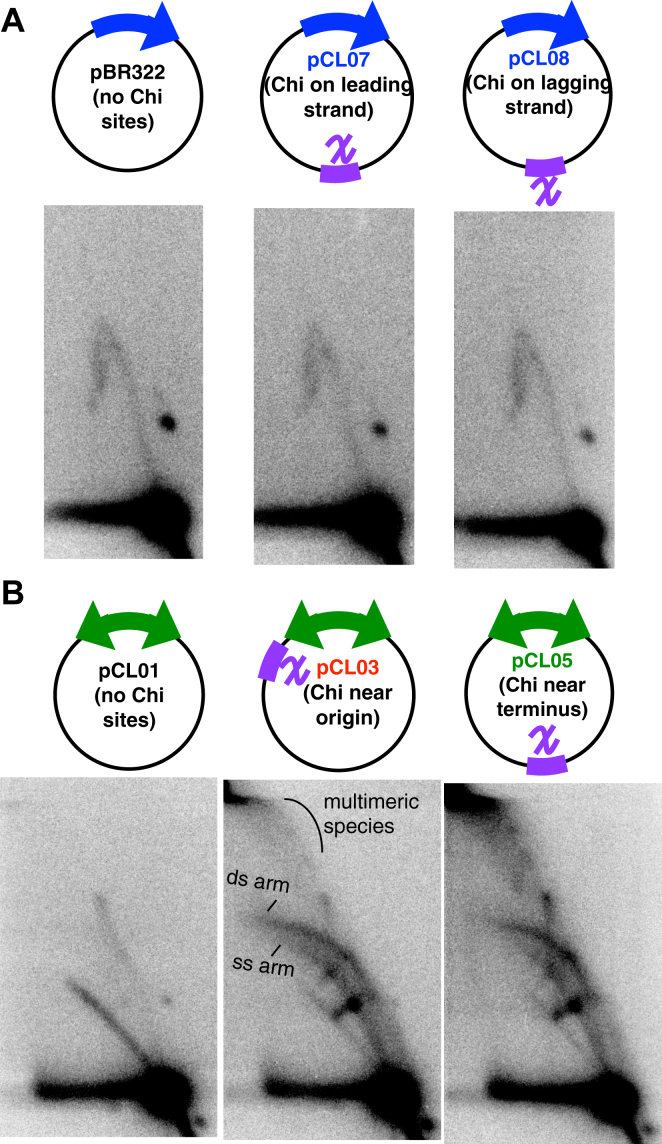
***chi* induces replication on substrates that contain convergent replication forks**. *A*, on plasmids containing one replisome, only the expected Y-shaped replication intermediates are observed, irrespective of whether *chi* is present or absent. Panel 1, no *chi*; panel 2, *chi* in the leading-strand template; panel 3, *chi* in the lagging-strand template. *B*, on plasmids containing two replisomes, replication initiation events leads to rolling circle replication when *chi* sequences are present. In the absence of *chi*, only the expected double Y-shaped replication intermediates are observed. However, in the presence of *chi*, prominent branched molecules having single- and double-strand DNA arms are observed, as well as multimeric fragments. Panel 1, no *chi*; panel 2, *chi* located proximal to the origin; panel 3, *chi* located opposite to the origin. (ds-arm) arc corresponding to branch molecules with double-strand DNA arm; (ss-arm) arc corresponding to branch molecules with single-strand DNA arm; (multimeric species) plasmid multimers containing single strand regions, resistant to restriction digestion. Total genomic and plasmid DNA was purified, digested with a restriction enzyme that cuts the plasmid at its origin of replication, and analyzed by 2D agarose gel analysis using ^32^P-labeled pBR322 (for A) or pCL01 (for B) as a probe.

Following 2D gel analysis of the one-replisome plasmids, we observed that *chi* sequences did not affect replication pattern, as replicating fragments formed a simple Y-arc, both in the presence and absence of *chi* (Fig. 4*A*). In the absence of *chi*, replicating fragments of two-replisome plasmids also formed the expected double-Y pattern (Fig. 4*B*). However, in the presence of *chi*, a broad arc representing the induction of rolling circle replication was observed. The arc contained species distributed between an upper and lower boundary, consistent with the presence of both single and double-strand DNA in the rolling circle DNA (46). Additionally, multimeric Y and double-Y shaped structures were also observed, with a significant amount of DNA failing to migrate into the gel. The presence of multimeric structures also implies that these intermediates contain significant amounts of single-strand DNA regions which prevent cleavage by the restriction enzyme. We would interpret these single strand regions to indicate that the replication occurring is uncoupled with respect to leading and lagging strand replication. Taken together, the observations demonstrate that RecBCD induces replication when encountering *chi* on structures created by convergent replication forks.

### *ter* sequences suppress *chi*-induced replication

**Figure 5:**
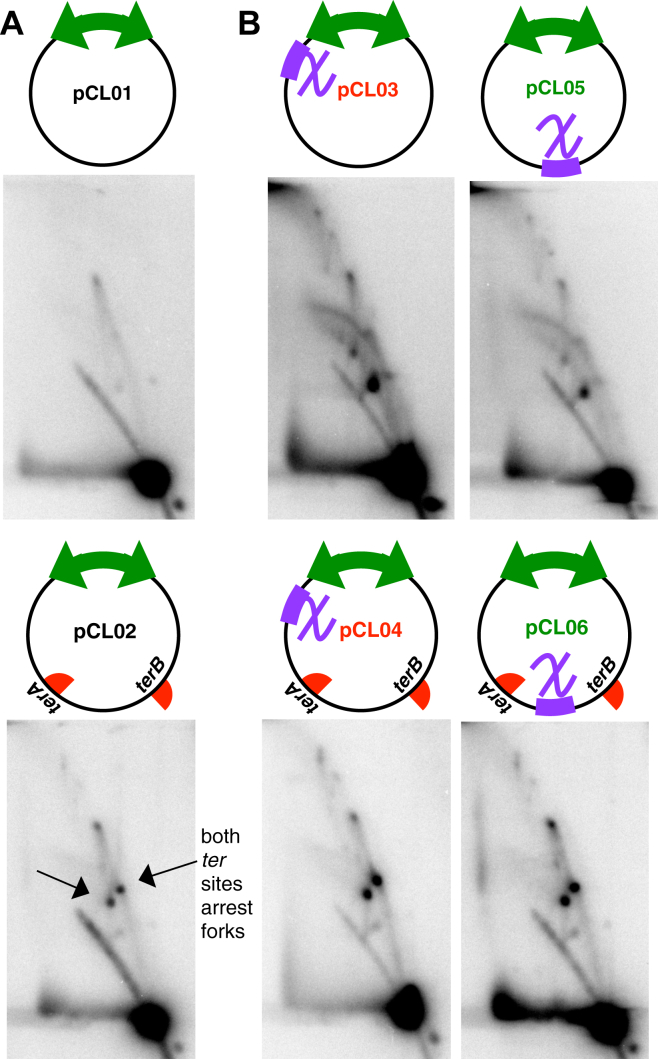
***ter* sequences suppress and limit the amount of *chi*-induced replication that occurs on plasmids containing convergent replication forks**. *A*, *ter* sequences arrest replisome progression on plasmids. Two prominent sites where the replisomes arrest, corresponding with the location of the *ter* sequences, are observed on plasmids containing *ter* sequences (*bottom panel*) but not in their absence (*top panel*). *B*, the presence of *ter* suppresses and limits *chi*-induced replication events. *Top panels*, *chi* induces replication events on plasmids with convergent forks, forming rolling circle replication and multimeric structural intermediates. *Bottom panels*, in the presence of *ter*, the *chi*-induced replication events are suppressed, while the replisome arrest sites remain. Arrest sites are indicated by *arrows*. DNA preparation and 2D agarose gel analysis was performed as in Figure 4.

On the chromosome, most completion events occur within a 400-kb region flanked by the termination sequences, *terA* and *terC*. The Tus protein binds *ter* sequences and inhibits replication fork progression in an orientation-dependent manner, in effect stalling the replication fork at these sites until the second replisome arrives (14, 17). To determine how these sequences affect *chi*-induced replication, we engineered two *ter* sequences into the two-replisome plasmid in an orientation that mimics that occurring on the chromosome. We then compared the replication patterns of these two-replisome plasmids in the absence and presence of a ‘*ter* trap’ by 2D agarose gel analysis. As shown in Figure 5*A*, the presence of *ter* sequences on the plasmid, pCL02, generated two arrest sites appearing as prominent spots on the replicating molecules. The observation confirms that both replisomes functionally initiate from the bidirectional origin and that both *ter* sequences actively arrest forks on these plasmids. When the *ter* sites were placed onto the two-replisome plasmids containing *chi* sequences, they partially suppressed the rolling circle replication and multimeric intermediates (Fig. 5*B*), indicating that arresting the forks at *ter* sites functionally limited the *chi*-induced replication.

### *ter* suppresses *chi*-associated instabilities during completion

**Figure 6:**
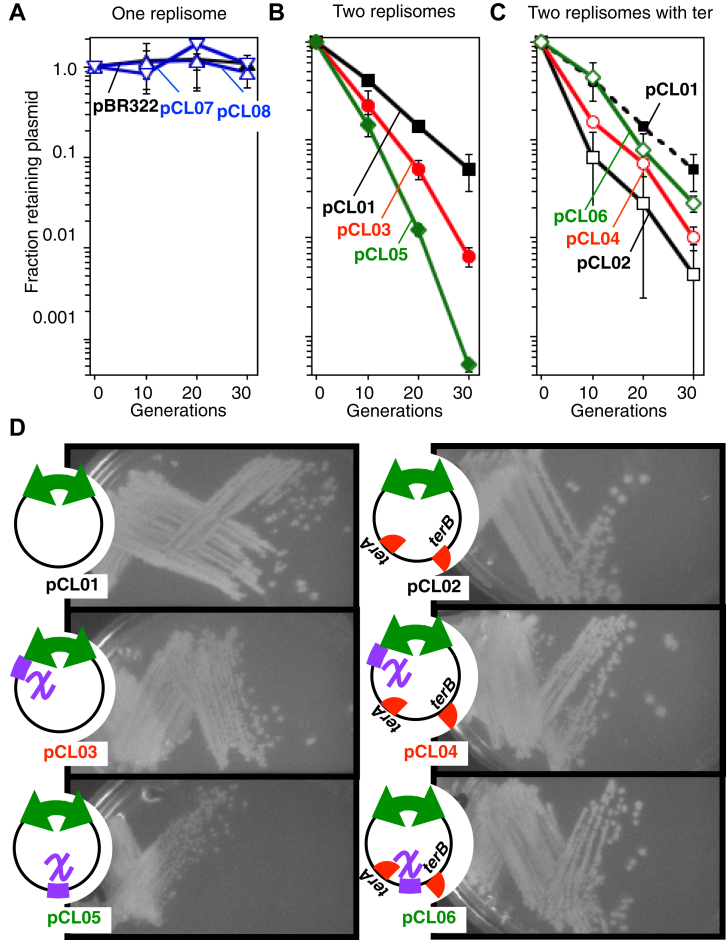
***ter* suppresses plasmid instability caused by *chi*-induced replication events**. *A*, the stability of plasmids containing one replisome are unaffected by the presence or absence of *chi*. *Filled triangle*, no *chi*; *open triangle*, *chi* in the leading-strand template; *open inverted triangle*, *chi* in the lagging-strand template. *B*, the presence of *chi* reduces the stability of plasmids containing two replisomes. *Filled square*, no *chi; filled circle*, *chi* proximal to origin; *filled diamond*, *chi* opposite to origin. *C*, in the presence of *ter*, the stability of *chi* containing plasmids improves. *open square*, no *chi* with *ter*; *open circle*, *chi* proximal to origin with *ter*; *open diamond*, *chi* opposite to origin with ter. *Filled square* no *chi*, from (*B*) is replotted for the purpose of comparison. Plots represent an average of two to four experiments. Error bars represent the standard error. *D*, *chi* sequences alter the morphology of colonies containing two-replisome plasmids, but the effect is suppressed in the presence of *ter*. The morphology of bacterial colonies containing two-replisome plasmids in the presence and absence of *chi* and *ter* is shown.

Excess replication can lead to amplifications and rearrangements that compromise stability on both the chromosome and plasmids. We therefore examined how *chi* affects the stability of the one-replisome and two-replisome plasmids. To this end, we monitored the fraction of cells retaining the plasmid during growth in nonselective media. Both the one- and two-replisome plasmids maintain similar copy numbers inside replicating cells (11). As shown in Figure 6*A*, the presence of *chi* sequences did not affect the stability of the one-replisome plasmid. However, on the two-replisome plasmid, the presence of *chi* further reduced plasmid stability (Fig. 6*B*). In addition, *chi* reduced the stability more when it was located opposite, rather than proximal, to the plasmid’s origin.

We also characterized the effect that *ter* sequences had on two-replisome plasmid stability both in the presence and absence of *chi*. The *ter* sequences by themselves were detrimental to the overall stability of the plasmid (Fig. 6*C*). However, *ter* sequences increased the stability of two-replisome plasmids containing *chi* sequences and correlated with the suppression of *chi*-induced replication events. The presence of *ter* increased the stability of the plasmids to a point that approximated that seen in the plasmids lacking *chi* sequence.

The effect of *chi* and *ter* also affected the morphology of the bacterial colonies in which they grew. Strains growing with plasmids that contained *chi* sequences were noticeably smaller (Fig. 6*D*), whereas the presence of *ter* sequences suppressed this effect, restoring the colonies to a size more similar to the non-*chi* plasmid colonies. Thus, *chi* appears to have a detrimental effect when it is encountered during the completion reaction. However, the presence of *ter* sequences oriented to trap replication suppresses the instabilities that arise when this occurs.

### The absence of RecD mimics the replicative state induced by encounters with *chi*

**Figure 7:**
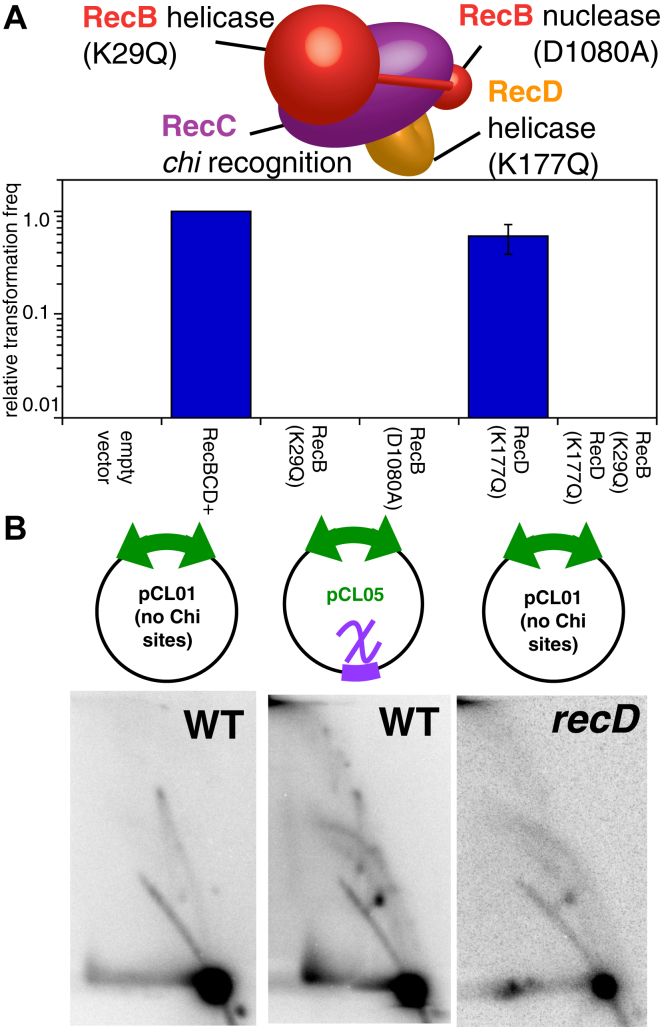
**The replication induced by *chi* requires the nuclease and helicase activity of RecB and is similar to that occurring in the absence of RecD.***A*, maintaining two replisome plasmids requires the nuclease and helicase and nuclease activity of RecB, but not the helicase activity of RecD. *recBCD* mutants expressing the indicated variants of RecBCD were transformed with the two-replisome plasmid, pCL01. The transformation frequency of each point mutant, relative to RecBCD+ culture, is plotted. Graphs represent the average of three experiments. Error bars represent the standard error of the mean. *B*, 2-D agarose gels are shown for a two-replisome plasmid replicating in wildtype cells (panel 1); a *chi*-containing two-replisome plasmid replicating in wildtype cells (panel 2); and a two-replisome plasmid replicating in a *recD* mutant (panel 3). Wildtype gels are reproduced from Figure 5 and shown for comparison.

The RecBCD complex contains dual ATP-dependent helicases that have opposite polarity and unwind DNA at different rates, as well as an exo/endonuclease that can act on either DNA strand and is regulated by the nonpalindromic 8 bp *chi* sequence, 5′ GCTGGTGG3′ (27, 48, 49) (Fig. 7*A*). To further characterize whether these enzymatic activities play a role in the completion reaction, we examined several point mutants lacking one or more of these activities. *recB*(D1080A) inactivates the nuclease activity in RecB. *recB*(K29Q) and *recD*(K177Q) are ATPase mutants that inactivate the helicase activities of RecB and RecD, respectively. We also examined the double helicase mutant *recB*(K29Q) and *recD*(K177Q). RecBCD complexes lacking either ATP-dependent helicase remain capable of unwinding DNA but do so differently than the wildtype. Complexes lacking both ATP-dependent helicases can bind, but not unwind DNA (27). *recBCD* deletion mutants expressing either the wildtype or mutant forms of RecBCD were examined for their ability to maintain plasmids with convergent replisomes. As expected, expression of wildtype RecBCD allowed cells to be transformed with the two-replisome plasmid, pCL01. However, complexes lacking the nuclease or helicase activity of RecB could not maintain the two-replisome plasmid. By contrast, complexes lacking the helicase activity of RecD remained proficient in maintaining the plasmid (Fig. 7*A*), similar to complexes deleted for RecD (8, 11). In the case of the *recBCD* deletion, *recB*(K29Q) and *recB*(D1080A) mutants, small pin-prick colonies could be observed after 48 h incubation. However, these colonies failed to grow further and could not be cultured. These results mimic those activities required during homologous recombination and indicate that the helicase and nuclease activities found in RecB are required, whereas the RecD subunit is not required but alters the reaction.

Complexes lacking RecD phenotypically mimic the RecBCD activity following encounters with *chi* in several ways. *chi* sequences attenuate the nuclease activity of the RecBCD complex, similar to that seen in the absence of RecD (32, 50, 51, 52). Additionally, *recD* mutations compromise the stability of both two-replisome plasmids and one-replisome plasmids in a manner that produces multimeric structures, suggesting an over-replication phenotype that mimics that seen in the terminus of the chromosome of *recD* mutants (8, 53, 54). Although we were unable to maintain two-replisome plasmids in *recB* or *recC* mutants, two-replisome plasmids can be transformed and grown in *recD* mutants. We therefore examined the intermediates forming on two-replisome plasmids in the absence of RecD. As shown in Fig. 7*B*, in *recD* mutants, rolling circle intermediates are observed in the two-replisome plasmid even in the absence of *chi*. The intermediates resembled those generated by *chi* sequences in wildtype cultures. The results demonstrate that the replication induced by *chi* at convergent forks derives from an attenuation of RecBCD nuclease activity, similar to that observed in the absence of RecD.

## Discussion

The data presented here show that *chi*, when encountered on completion substrates, switches the activity of RecBCD from promoting degradation to promoting replication. The *chi*-induced replication is specific to substrates that contain convergent replisomes and is detrimental to completion and genomic stability overall. However, chromosomal *ter* sequences functionally suppress the instability associated with the *chi*-induced replication (Figs. 5 and 6).

**Figure 8:**
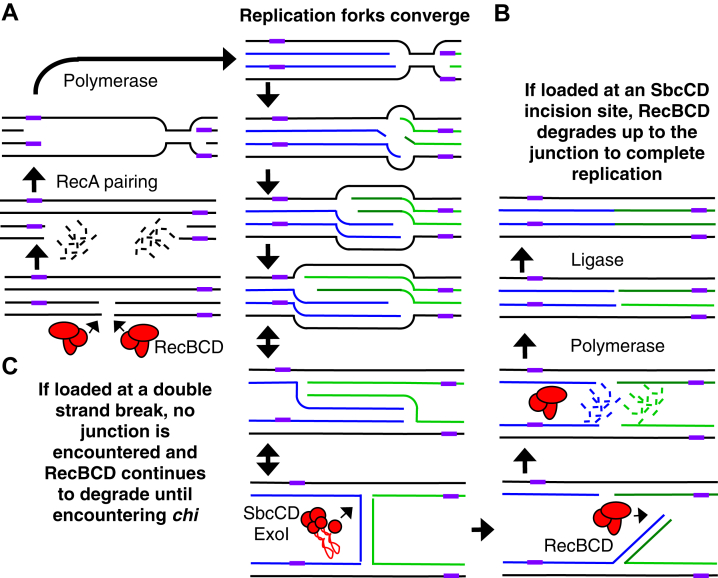
**Model for *chi* and *ter* function during completion and double-strand break repair**. *A*, during the completion of DNA replication, replication forks transiently bypass each other, creating a limited region of over-replicated DNA that has a pseudo-palindrome-like structure which can be cleaved by SbcCD and ExoI structure-specific nucleases. *B*, RecBCD binds to the double-strand end created by these nucleases and promotes degradation of the excess DNA, arresting at the branch point. Polymerase and ligase then complete replication. *C*, when RecBCD is loaded at a double-strand break, degradation continues until a *chi* site (*purple line*) is encountered. This promotes RecA-mediated pairing with homologous duplex DNA and polymerase recruitment, leading to replication at these sites.

What functional purpose could *chi* serve? One possibility is that *chi* serves to functionally differentiate DNA ends created during completion, which require degradation, from those created by chromosomal double-strand breaks, which require resynthesis. During the completion of replication, the processivity of the replisome’s helicases allows replication forks to bypass each other, creating a limited region of over-replicated genetic material [*i.e.*, a third copy at sites where replication forks converge (4, 8, 9, 10) (Fig. 8*A*)]. Completion is thought to initiate through the action of structure-specific nucleases, SbcCD and Exo I, which incise the palindrome-like structure created at the over-replicated region (10, 11). This creates a double-strand DNA end on the third copy of genetic material, providing a substrate for RecBCD to enter, unwind, and promote degradation. Once the over-replicated region is degraded, and the branch point is reached, RecBCD may recruit or lead to recruitment of a polymerase to fill in the remaining gap before ligase joins the nascent strands (Fig. 8*B*). *In vitro*, RecBCD degradation arrests at the base of cruciform DNA. It is unknown if unwinding continues past this point (55). Assuming that the over-replicated region is limited, RecBCD would not encounter a *chi* sequence, which arises on average every 4 to 5 kb on the genome, during most completion events. In those rare situations where RecBCD does encounter *chi* while degrading excess DNA, the presence of *ter* may limit the extent to which these illegitimate replication events can occur, thereby allowing RecBCD to reload and degrade them.

Alternatively, in the case where a double-strand break arises in the chromosome, RecBCD unwinding and degradation would continue without encountering a junction until a *chi* sequence is encountered. In the presence of RecA, pairing with homologous duplex DNA would convert the structure to one which resembles that created during the completion of replication (Fig. 8*C*). Similarly, if a one-ended break was produced (not shown in model), pairing and recruitment of the replisome would simply allow replication to resume as has been proposed previously (56). Induction of a double-strand break has previously been shown to induce DNA replication in a manner that depends on RecBCD and a properly oriented *chi* site (57).

This type of model would be consistent with several of the observations shown here. Two-replisome but not one-replisome plasmids require RecBCD to be maintained (Fig. 1 and (11)), demonstrating that convergent replication forks create a substrate that requires RecBCD processing. *chi* is not essential to maintain two-replisome plasmids consistent with the idea that this sequence is not required and may not normally play a role in RecBCD processing at intermediates created by convergent forks. However, addition of *chi* induces replication on these substrates and leads to multimeric amplifications that are detrimental to stability and the completion reaction (Figure 2, Figure 4, Figure 6). Additionally, the addition of *ter* sequences, limited the amount of *chi*-induced replication and improved plasmid stability (Figs. 5 and 6).

The model is also consistent with the observations that RecA is not required to complete replication on the chromosome or plasmids but is required for double-strand break-induced replication and repair [Fig. 1 (8, 9, 11, 57, 58, 59, 60)]. Additionally, *chi* alters RecBCD activity in a manner that promotes RecA recruitment and loading at these substrates (61, 62), suggesting an ability of *chi* to recruit polymerase and induce replication that may be coupled with RecA loading (63, 64). Consistent with this, RecA is required for the multimeric amplification of plasmids driving their instability (11, 53, 65, 66). Similarly on the chromosome, aberrant extensive over-replication that occurs in mutants impaired in their ability to complete replication also requires the presence of RecA (2, 8, 10).

In the case of *recD* mutants, we observe intermediates similar to those seen in the presence of *chi*, suggesting that the RecBC complex is in a form that promotes polymerase recruitment even in the absence of *chi*. Consistent with this, RecBC complexes lacking RecD appear constitutive for RecA loading (67), and *recD* mutants exhibit instabilities and generate multimeric species on plasmids as well as over-replicate the terminus region of the chromosome (8, 53, 66).

The polymerase or replisome component recruited to *chi* substrates by RecBCD is not currently known. Early studies suggested polymerase I may associate with RecBCD in a large complex (68, 69, 70). However, if significant gaps remain on completion substrates following RecBCD processing, more processive polymerases associated with DNA sliding clamps may be involved. We would speculate that the recruitment would not be associated with novel loading of the replicative helicase, which is thought to be responsible for the over-replication (2, 8). A processive polymerase, perhaps loaded by the gamma complex seems likely given the extensive replication induced by *chi* (71). The multimeric over-replicated species observed on two-replisome plasmids with *chi* are partially resistant to restriction digestion suggesting that they contain significant regions of single-stranded DNA. This would be consistent with processive replication that is uncoupled and lacking coordination of the associated replicative helicase by tau complex (71).

Processive replication is also likely to be required when a double-strand break arises on the chromosome. Following RecBCD processing, extensive resynthesis of the degraded sequences would be required. One-ended double-strand breaks are also frequently speculated to arise when replication encounters a nick on the leading strand. In this case, reloading a processive polymerase would also be necessary. Re-association with the pre-existing helicase on the DNA may then be sufficient to restore a processive replisome. One-ended breaks at ongoing forks would also explain why *chi* sequences are found heavily biased in the leading-strand template (9, 42, 72), since processing would only occur on this strand.

Following DNA damage-induced stress, the completion reaction is transiently inhibited during the recovery period, leading to over-replication of the region where forks converge. Similar to what was observed on the plasmids, the over-replicated region is limited by *ter* sequences (73). Interestingly, mutants specifically impaired in their ability to complete replication, such as *recG*, *recBCD*, *sbcCDxonA*, create a bottleneck at this point in the cell cycle, producing an unbalanced amplification of the rest of the genome as new initiations continue to occur (73).

Several completion enzymes are highly conserved between *E. coli* and humans (74, 75, 76). Further, amplifications and instabilities similar to those seen in the terminus region of *E. coli* are commonly observed in human cancers (23, 77, 78, 79, 80, 81, 82). Unlike *E. coli*, completion occurs thousands of times each generation throughout the chromosomes of human cells, arguing that completion could be a large and underappreciated source driving instabilities in these cells.

## Experimental procedures

### Strains and plasmids

The parental strain used in this work is BW25113 of genotype *rrnB3* Δ*lacZ*4787 *hsdR*514 Δ(*araBAD*)567 Δ(*rhaBAD*)568 *rph*-1 and is the parental strain used to construct the Keio single gene knockout mutant library (83). JW2669, JW2788, and JW2787 are *recA*::FRT-kan-FRT, *recB*::FRT-kan-FRT, and *recD*::FRT-kan-FRT derivatives of BW25113, respectively (Baba *et al*., 2006). CL4518, CL4570 and CL4606 are *xonA*::FRT-kan-FRT *sbcCD*::gent, *xonA*::FRT *sbcCD*::gent and *xonA*::FRT *sbcCD*::gent *recA*:FRT-kan-FRT derivatives of BW25113, respectively. CL4518 was made by P1 transduction of *sbcCD*::gent from KM135 (84) into JW1993 (83), selecting for gentamicin resistance. CL4570 was made by FLP-mediated loss of the kanamycin cassette from strain CL4518 using plasmid pCP20 (85) as described in (86). CL4606 was made by P1 transduction of *recA*::FRT-kan-FRT from JW2669 into CL4570, selecting for kanamycin resistance. CL5250 was made by P1 transduction of *recBCD*::kan from KM21 (87) into BW25113, selecting for kanamycin resistance.

Plasmid constructions were performed according to published protocols for PCR amplification, Gibson assembly, and *in vivo* recombineering (88, 89). Plasmids containing unidirectional origins of replication were derived from pBR322, have ampicillin- and tetracycline-resistance cassettes, and utilize a pMB1 origin of replication derived from ColE1 (11, 90). pCL07 and pCL08 contain a *chi* sequence engineered into the leading and lagging strand of pBR322, respectively. Primer pairs 5′-catgcccggttactggaacggctggtggttgtgagggtaaacaactgg-3'/5′-cgccgcatacactattctca-3' for pCL07 and 5′- ccagttgtttaccctcacaaccaccagccgttccagtaaccgggcatg-3'/5′-tgagaatagtgtatgcggcg-3′ for pCL08 were used with pBR322 as a template and amplified for 25 cycles using Pfu Turbo Polymerase (Agilent). PCR products were purified by agarose gel electrophoresis, combined with DpnI digested pBR322, and then joined and transformed using Gibson assembly (New England Biolabs) to generate pCL07 and pCL08. Plasmids were sequenced to verify sequence changes.

Plasmids containing bidirectional origins of replication are derived from pCB104 and contain a bidirectional origin of replication from phage lambda and chloramphenicol- and ampicillin-resistance cassettes (11, 91). Primers 5ʹgtcggttcagggcagggtcgtggatccactttagttacaacatacttattcgcggaacccctatttgttt and 5ʹggcggtttgcgtattgggcgcatattagttacaacatcctatatggtctgacagttaccaatgc were used to amplify the ampR gene from pBR322. 0.2 μg gel-purified PCR product was then combined with 0.5 μg BamHI-digested pCB104 and amplified for 25 cycles using Pfu Turbo Polymerase (Agilent). PCR products larger than 5kb were purified by agarose gel electrophoresis and transformed into recombineering strain DY329 (Yu *et al*., 2000) to generate plasmid pCL03, which contains a *chi* site proximal to the origin.

The *chi* site of pCL03 was removed using two primer pair sets 5′-attgctgataaatctgga-3’/5′-ctttggaatccagtccctcttcctcctgctgatctgcgacttatcaac-3′ and 5′-tccagatttatcagcaat-3’/5′-gttgataagtcgcagatcagcaggaggaagagggactggattccaaag-3′ to amplify overlapping fragments of the plasmid template using Pfu Turbo Polymerase (Agilent). The fragments were then joined and transformed using Gibson assembly (New England Biolabs) to generate pCL01.

pCL05 was constructed by inserting a *chi* sequence into the terminus region of plasmid pCL01, using plasmid pairs 5′-ctgcgctcggcccttccggctgccaccagcattgctgataaatctgga-3'/5′-tccagatttatcagcaatgctggtggcagcggaagggccgagcgcag-3' and 5′-gttgataagtcgcagatcagcaggaggagaagagggactggattcc-aaag-3'/5′-ctttggaatccagtccctcttcctcctgctgatctgcgacttatcaac-3' to amplify overlapping fragments which were joined and transformed using Gibson assembly (New England Biolabs).

pCL02, pCL04, and pCL06 are identical to pCL01, pCL03, and pCL05 but contain *terB* and *terC* sequences that flank the ampR cassette. pCL04 was constructed using primer pairs 5′-gtcggttcagggcagggtcgtggatccactttagttacaacatacttattcgcggaacccctatttgttt-3′and 5′-ggcggtttgcgtattgggcgcatattagttacaacatcctatatggtctgacagttaccaatgc-3′to amplify the ampR cassette from pBR322. 0.2 μg gel purified PCR product was then combined with 0.5 μg BamHI-digested pCB104 and amplified for 25 cycles using Pfu Turbo Polymerase. PCR products larger than 5kb were gel purified and transformed into recombineering strain DY329 (92) to generate the ampicillin-resistant plasmid.

Primer pairs 5′-ctgcgctcggcccttccggctgccaccagcattgctgataaatctgga-3' +5′-tccagatttatcagcaatgctggtggcagcggaagggccgagcgcag-3' and 5′-gttgataagtcgcagatcagcaggaggagaagagggactggattccaaag-3' and 5′-ctttggaatccagtccctcttcctcctgctgatctgcgacttatcaac-3' were used to amplify overlapping fragments of the pCL04 template that were joined and transformed using Gibson assembly (New England Biolabs) to generate pCL02 and pCL06. (see Table 1).

**Table 1:** Strains and plasmids

Strains	Reference	Relevant genotype
BW25113 Parent	(86)	(*araD*-*araB*)567, Δ*lacZ4787*(::*rrnB*-3), λ-, *rph*-1, Δ(*rhaD*-*rhaB*)568, *hsdR*514
JW2669	(83)	BW25113 *recA*::FRT-kan-FRT
JW2788	(83)	BW25113 *recB*::FRT-kan-FRT
CL4518	This study	BW25113 *xonA*:: FRT-kan^R^-FRT *sbcCD*::gent^R^
CL4570	This study	BW25113 *xonA*::FRT *sbcCD*::gent^R^
CL4606	This study	BW25113 *xonA*::FRT *sbcCD*::gent^R^*recA*:FRT-kan^R^-FRT
CL5250	This study	BW25113 *recBCD*::kan
JW2787	(83)	BW25113 *recD*::FRT-kan-FRT
KM135	(84)	*sbcCD*::gent^R^

Plasmids	Reference	Relevant properties

pBR322	(90)	*amp*^R^*tet*^R^ ColE1 origin
pCL01	(11)	*amp*^R^*cam*^R^ λ origin
pCL02	This study	pCL01 with *terA terC*
pCL03	This study	pCL01 with *chi* proximal to *ori*
pCL04	This study	pCL01 with *terA terC* and *chi* proximal to *ori*
pCL05	This study	pCL01 with *chi* opposite to *ori*
pCL06	This study	pCL01 with *terA terC* and *chi* opposite to *ori*
pCL07	This study	pBR322 with leading strand *chi*
pCL08	This study	pBR322 with lagging strand *chi*
pCP20	(85)	*amp*^R^*cam*^R^ temperature-sensitive replication. thermal induction of FLP synthesis.
pSA607	(39)	pBR322 containing operon *recBrecC*(6xhis)*recD*
pSA335	(39)	pBR322 containing operon *recB*(D1080 A)*recC*(6xhis)*recD*
pSA618	(39)	pBR322 containing operon *recB*(K29Q)*recC*(6xhis)*recD*
pSA620	(39)	pBR322 containing operon *recBrecC*(6xhis)*recD*(K177Q)
pSA622	(39)	pBR322 containing operon *recB*(K29Q)*recC*(6xhis)*recD*(K177Q)

### Replication profiles

Cultures grown overnight were diluted 1:250 in fresh LB media. All cultures were grown at 37 ºC with aeration, unless otherwise indicated. To normalize profiles, stationary-phase cultures were grown for 36 h before harvesting. When cultures reached an *A*_600_ of 0.4, genomic DNA was purified by placing 0.75-mL of culture into 0.75-mL ice-cold 2X NET buffer (100 mM NaCl, 10 mM Tris at pH 8.0, 10 mM EDTA). All samples were then pelleted by centrifugation, resuspended in a solution containing 140 μl of 1 mg/ml lysozyme and 0.2 mg/ml RNaseA in TE (10 mM Tris at pH 8.0, 1 mM EDTA), and incubated at 37 °C for 30 min to lyse cells. Subsequently, Sarkosyl [10 μl, 20% (wt/wt)] and proteinase K (10 μl, 10 mg/ml) were added, and the incubation was continued at 37 °C for an additional 30 min. The samples were then further purified by extracting the DNA with 4 vol phenol/chloroform (1/1) followed by dialysis for 1 h using 47 mm MF-Millipore 0.05-μm pore disks (#VMWP04700; Merck Millipore) to float the samples on a 250-mL beaker of TE buffer (1 mM Tris at pH 8.0, 1 mM EDTA).

Genomic DNA samples were sequenced using paired-end, 51-bp, bar-coded reads prepared and run using seqWell library prep kits (seqWell) and Illumina Next Seq 2000 (Illumina) following the manufacturer’s instructions. Gene mutations in each strain were confirmed using the program Breseq to identify differences with the reference genome for BW25113 (93). For all strains, the original Illumina sequence reads were aligned to the BW25113 reference genome and assembled using the program Bowtie 1.0.0 (94). All aligned reads were then characterized to determine the nucleotide frequency at each position. The number of sequences per kilobase was determined and plotted using a custom Python script. To prevent sequencing bias caused by the purification or sequencing, the copy number for each strain was normalized to a stationary-phase culture of BW25113. Plots represent these relative copy number values at each genomic location in 1-kb bins and depict the replication profile of each strain.

### Plasmid transformation

Electrocompetent cells were prepared by growing a 100-fold dilution of a fresh overnight culture in 10 ml LB to an *A*_600_ of 0.4. Cells were pelleted, serially washed with 30 ml water, 30 ml 10% glycerol, resuspended in 200 μl of 10% glycerol, and stored at −80 °C. To determine transformation efficiency, 40 μl of competent cells were mixed with a plasmid mixture containing both pBR322 and pCL01, electroporated at 2.5 kV with capacitance of 25 μFD and resistance of 200 Ω, and allowed to recover at 37 °C for 30 min in 1-ml SOC media. The transformation reactions were then serially 10-fold diluted and triplicate 10-μl aliquots of each dilution were spotted on three sets of LB plates containing no additions, 15 μg/ml tetracycline, or 20 μg/ml chloramphenicol to determine the number of viable cells and transformants for each plasmid, respectively. Colonies were counted following overnight incubation at 37 °C, unless otherwise indicated. The same mixture of plasmid DNA was used for all strains. The relative transformation efficiency of each strain was calculated as the ratio of transformants per viable cells in the mutant cultures to the transformants per viable cells in wildtype cultures.

### Plasmid stability

Plasmids were transformed into cells by electroporation. Cells from overnight cultures of strains containing the plasmid grown in LB medium with 50 μg/ml ampicillin were pelleted and used to inoculate 10 ml cultures of LB medium at 1:1000 dilution. Cultures were grown without ampicillin selection at 37 °C with aeration overnight. The resulting cultures were then sampled to determine the ratio of cells retaining the plasmid and used to reinoculated 10 ml LB medium at 1:1000 dilution. This was repeated for three iterations. To determine plasmid retention, triplicate 10-μl aliquots of serial 10-fold dilutions were spotted on LB plates in the presence or absence of 50 μg/ml ampicillin. Colonies were counted following overnight incubation at 37 °C (Wendel *et al*., 2014).

### Total genomic and plasmid DNA purification

Overnight cultures of cells containing the plasmid were diluted 1:100 in LB medium containing 100 μg/ml ampicillin and grown in a 37 °C shaking water bath to an *A*_600_ of 0.5. Seven hundred fifty microliter of cultures was mixed with 750 μl of ice-cold 2× NET (100 mM NaCl, 10 mM Tris, pH 8.0, 10 mM EDTA). Cells were pelleted and frozen at −80 °C. Samples were resuspended in 140 μl of lysozyme (1 mg/ml) and RNaseA (0.2 mg/ml) in TE (10 mM Tris, pH 8.0, 1 mM EDTA) and lysed for 30 min at 37 °C. Then Sarkosyl (10 μl of 20% [wt/wt]) and Proteinase K (10 μl of 10 mg/ml) was added and incubation continued for 60 min. Samples were then serially extracted with two volumes phenol then chloroform (1/1) and then again with two volumes chloroform followed by dialysis for 1 h on 47 mm Whatman 0.05-μm pore disks (Whatman #VMWP04700) which were floated on a 250-mL beaker of TE (1 mM Tris, pH 8.0, 1 mM EDTA).

### 1-D agarose gel analysis of plasmid replication intermediates

Total genomic DNA was digested with SacII (New England BioLabs) for strains containing pBR322-derived plasmids or NheI (New England BioLabs) for pCB104-derived plasmids. In both cases, plasmids lack restriction sites for these enzymes. Samples were then extracted with one volume of chloroform before equal cell equivalents were electrophoresed through 1.0% agarose gel in 1× TBE (Tris-borate-EDTA, pH 8.0) at 1 V/cm. DNA in the gels was transferred to a Hybond N+ nylon membrane, and the plasmid DNA was detected by probing with either ^32^P-labeled pBR322 or pCL01 plasmid DNA prepared by random-primer labeling (Agilent Technologies) using α^32^P-labeled-dCTP (3000 Ci/mmol; PerkinElmer) and visualized using a STORM PhosphorImager with its associated ImageQuant analysis software (Amersham Biosciences).

### 2-D agarose gel analysis of plasmid replication intermediates

Total genomic DNA was digested with PvuII (New England BioLabs) for strains containing pBR322-derived plasmids, or BstEII (New England BioLabs) for pCB104-derived plasmids. In both cases, these enzymes restrict the plasmid near its origin of replication. For the first dimension, samples were extracted with one volume of chloroform before equal cell equivalents were electrophoresed in a 0.4% agarose gel in 1× TBE at 1 V/cm for 15 h. For the second dimension, the lanes were excised, rotated 90°, and recast in a 1% agarose gel in 1× TBE and electrophoresed at 6.5 V/cm for 6.5 h. Southern analysis was carried out as described above for 1-D agarose gel analysis.

## Data availability

All data are included in the article or are available from the corresponding author J.C. Strains and plasmids used in this study are available upon request.

## Conflict of interest

The authors declare no conflict of interest with the contents of this article.
